# Nocturnal flight-calling behaviour predicts vulnerability to artificial light in migratory birds

**DOI:** 10.1098/rspb.2019.0364

**Published:** 2019-04-03

**Authors:** Benjamin M. Winger, Brian C. Weeks, Andrew Farnsworth, Andrew W. Jones, Mary Hennen, David E. Willard

**Affiliations:** 1 Museum of Zoology, Department of Ecology and Evolutionary Biology, University of Michigan, 1105 North University Avenue, Ann Arbor, MI 48109, USA; 2 Cornell Laboratory of Ornithology, 159 Sapsucker Woods Road, Ithaca, NY 14850, USA; 3 Department of Ornithology, Cleveland Museum of Natural History, 1 Wade Oval Drive, University Circle, Cleveland, OH 44106, USA; 4 Gantz Family Collections Center, The Field Museum, 1400 South Lake Shore Drive, Chicago, IL 60605, USA

**Keywords:** nocturnal migration, flight calls, artificial light at night, building collisions, collective migration

## Abstract

Understanding interactions between biota and the built environment is increasingly important as human modification of the landscape expands in extent and intensity. For migratory birds, collisions with lighted structures are a major cause of mortality, but the mechanisms behind these collisions are poorly understood. Using 40 years of collision records of passerine birds, we investigated the importance of species' behavioural ecologies in predicting rates of building collisions during nocturnal migration through Chicago, IL and Cleveland, OH, USA. We found that the use of nocturnal flight calls is an important predictor of collision risk in nocturnally migrating passerine birds. Species that produce flight calls during nocturnal migration tended to collide with buildings more than expected given their local abundance, whereas those that do not use such communication collided much less frequently. Our results suggest that a stronger attraction response to artificial light at night in species that produce flight calls may mediate these differences in collision rates. Nocturnal flight calls probably evolved to facilitate collective decision-making during navigation, but this same social behaviour may now exacerbate vulnerability to a widespread anthropogenic disturbance. Our results also suggest that social behaviour during migration may reflect poorly understood differences in navigational mechanisms across lineages of birds.

## 1. Introduction

The nearest that we can usually come to observing the flight itself is to stand out in the open on some starlit night and listen to the faint chirps that come floating down from the great vault above. We strain our eyes in an effort to catch a glimpse of the throng that we know must be passing overhead, but all in vain, for the migrants of the night are shielded by the darkness alike from friend and foe. (Stone 1906 [1, p. 249]).

Information derived from social cues is considered a critical component of animal migration, aiding in navigational decisions and in selecting stopover habitat [2–5]. The ‘many wrongs’ hypothesis posits that minor errors made by individuals during their movements may be corrected by group cohesion, such that collectively the group will make proper navigational decisions [2,6,7]. However, our empirical understanding of collective migratory behaviour is lacking, particularly among species whose social behaviour is not easily observed during migration. Nowhere is this knowledge gap starker than in nocturnally migrating species that are hidden from human view during their passage [8,9]; indeed, one of the greatest barriers to researching bird migration is that many bird species migrate at night, making direct observation of migrating individuals difficult. Technologies such as radar [10,11], ceilometers [8], thermal imaging cameras [12] and tracking tags [13,14] have provided a wealth of information on the behaviour of nocturnally migrating birds, such as insights into the density, direction and speed of migration. However, these technologies either do not provide the ability to distinguish among species or, in the case of tracking tags, to generalize beyond a small number of individuals, limiting our ability to test hypotheses on the social biology of nocturnal migration.

One of the few means to examine species-specific dynamics of social biology during nocturnal bird migration is through the study of short vocalizations made in flight by migrating birds (electronic supplementary material, figure S1). Many species of birds, especially passerines (order Passeriformes), produce such vocal signals during their nocturnal migrations [15]. These calls (hereafter, ‘flight calls’) are hypothesized to function as important social cues for migrating birds that may aid in orientation, navigation and other decision-making behaviours [15–23]. However, the difficulty of observing nocturnal migration has impeded progress in linking flight calls to specific migratory decisions or strategies [15]. Consequently, the extent to which nocturnally migrating birds rely on information from individuals vocalizing in proximate airspace to guide their navigational decisions is unknown [4,24] and our understanding of the social function of flight calls remains speculative. Moreover, not all nocturnally migratory species make flight calls, raising the possibility that different lineages of migratory birds vary in the degree to which social cues and collective decisions are important for accomplishing migration. For example, among North American passerine birds, flight calls are commonly given by thrushes, some nine-primaried oscines (e.g. New World warblers and sparrows) and others, but are largely absent in other nocturnally migrating taxa such as tyrant flycatchers and vireos [16,19].

In this paper, we investigate the importance of flight calls as potential social cues for collective decision-making among nocturnally migratory passerine birds by examining the relationship between flight calling and behavioural responses to a disruptive stimulus: artificial (i.e. anthropogenic) light at night (henceforth, ‘artificial light’). Artificial light exerts powerful, disruptive and widespread effects on nocturnally migrating birds and other organisms through phototaxis, in which birds are drawn towards light sources [1,25–34]. Mortality may result from such disruptions when birds collide with illuminated structures or become attracted to, and ‘trapped’ in, dangerous areas [26,28,29,35–37]. Previous studies have demonstrated that rates of nocturnal flight calling in passerine birds increase during migratory passage over areas with artificial light relative to unlit areas [27,28,34]. These observations suggest that stimulation from, or disorientation by, artificial light may escalate the use of social cues that birds rely on during their nocturnal migrations. We hypothesized that if flight calls are important social cues for decision-making during nocturnal migration, individuals from species that make flight calls may attract one another vocally when disoriented by artificial light. This relationship may spawn a vicious cycle of increased mortality rates if disoriented individuals lead other migrating individuals to sources of artificial light. However, the consequences of artificial light-induced increases in flight calling for migratory navigation and decision-making are poorly understood, as are the impacts on mortality.

**Figure 1.**
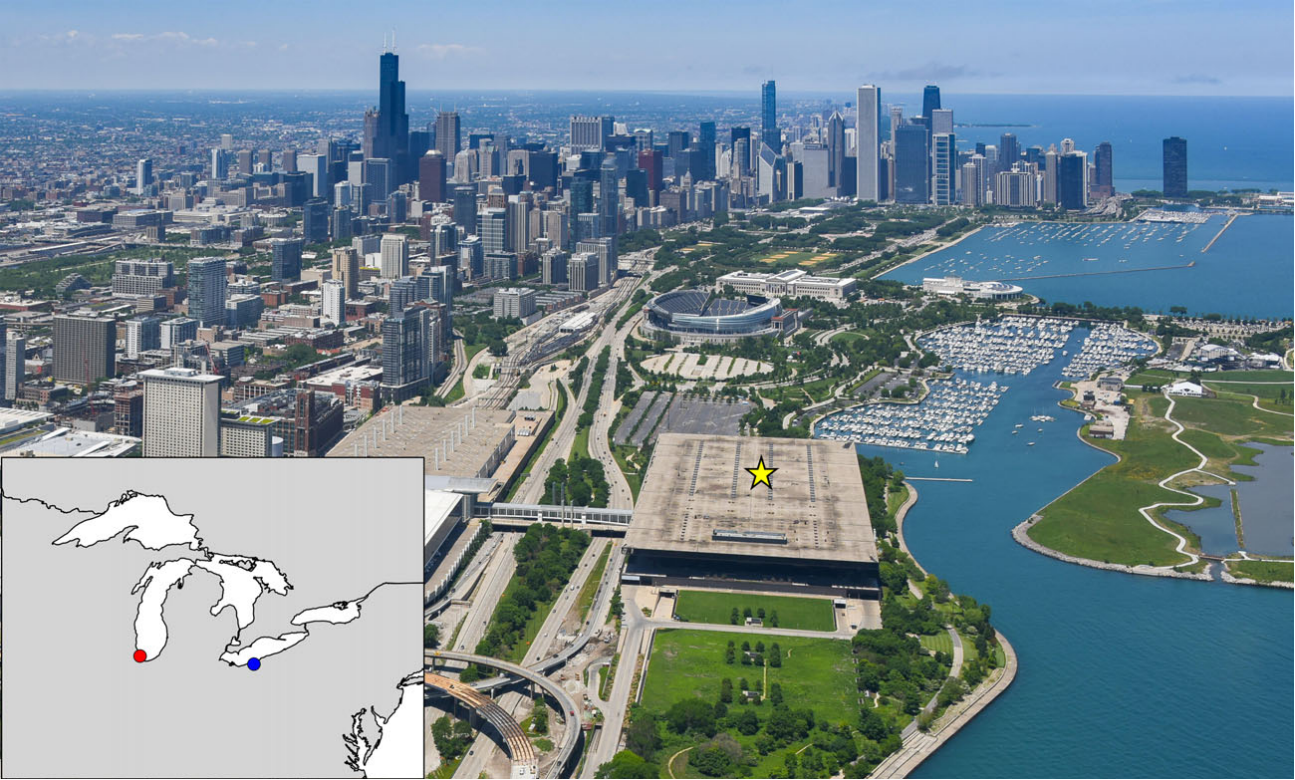
Study locations. Inset map: Our study examined records of lethal building collisions of passerine birds from Chicago, IL, USA (red dot) from 1978 to 2016 (69 785 collision records) and Cleveland, OH, USA (blue dot) in 2017 (2229 collisions). Photo: Chicago, IL study locations. More than half of the Chicago collision records were collected around the perimeter of McCormick Place convention centre (yellow star; see also the electronic supplementary material, figure S2). The remaining specimens were gathered throughout downtown Chicago (background). Photo courtesy of Curtis Waltz. (Online version in colour.)

We leveraged data on interspecific variation in the production of flight calls, coupled with a 40-year study of bird collisions with buildings in Chicago, IL, USA, to test how flight calling influences species responses to artificial light. The dangers of artificial light for migratory birds are particularly acute in densely lit urban areas located along migratory flyways [36,37]. Not only does artificial light result in mortality from direct collisions with illuminated buildings at night, but it can cause widespread indirect mortality when migrating birds that were initially attracted by light to inhospitable areas during the night collide with reflective glass the next day while searching for proper habitat [36]. A recent study using remote-sensing revealed that illuminated buildings in Chicago collectively expose the largest number of migratory birds to the highest levels of artificial light relative to all other cities in the USA [38]. Thus, records of building collisions in Chicago provide a strong proxy for measuring the disruptive effects of artificial light on different species. Although we focused our analyses on bird collision frequencies from Chicago because of the large size of this collision dataset (see Material and methods), we also compared our results to a smaller dataset from Cleveland, OH, located 500 km east of Chicago on the south shore of Lake Erie, to test the robustness of our conclusions across sites (figure 1).

Based on our hypothesis that nocturnal flight calls exacerbate attraction to artificial light, we predicted higher collision mortality rates for species that make nocturnal flight calls than closely related species that do not regularly produce nocturnal flight calls. In our analyses, we controlled for phylogenetic non-independence of species, local relative abundance and regional population size, as well as habitat differences among species that could influence their response to artificial light. For example, habitat differences have been linked to variation in the visual perception of migratory warblers [39], raising the possibility that the light environment a species typically inhabits may select for visual adaptations that could interact differently with artificial light. To further test the relationship between artificial light and building collisions across species with different behavioural ecologies, we analysed a 19-year dataset on the extent of night-time lighting at a single building that is known to be dangerous for migratory birds, the McCormick Place convention centre in Chicago. The lakefront location of McCormick Place, along with its large recessed windows and practice of sometimes keeping lights on at night, evidently (figure 1; electronic supplementary material, figure S2) makes nocturnally migrating birds vulnerable to attraction and collision, despite the fact that the building is not a high rise (see Results). We predicted that if flight calling influences overall collision risk during nocturnal migration through Chicago and Cleveland, then levels of artificial light at McCormick Place should affect the collision rates of species differentially based on this aspect of their social biology. Our records of the extent of artificial light at McCormick Place thus serve as a more direct test of patterns suggested by overall collision frequencies throughout our broader study region.

Our examination of the influence of social cues on rates of building collisions represents one of the first comparative studies of avian social behaviour during nocturnal migration. Our findings reveal the potential for broadly different navigational strategies among closely related lineages of nocturnally migratory birds depending on their social biology, and highlight how differences in species' behavioural ecologies and natural histories interact with anthropogenic threats in unexpected but consequential ways.

## 2. Material and methods

### (a) Collision sampling

D.E.W., M.H. and other Field Museum personnel monitored nocturnal bird collisions at McCormick Place (figure 1; electronic supplementary material, figure S2) in Chicago, IL by walking around the building every morning during spring (March through to May) and autumn (late August through to November) migration from 1978 to 2016. Starting in 2002, the Chicago Bird Collision Monitors, a volunteer network, began coordinating a collision monitoring effort at other buildings throughout downtown Chicago for the purpose of increasing awareness and understanding of bird collisions; prior to 2002, specimens from throughout Chicago were salvaged occasionally. Collision monitoring began in spring 2017 in downtown Cleveland, OH by a community organization called Lights Out Cleveland; our analyses include data from autumn 2017 and spring 2018. Carcasses from lethal collisions were brought to The Field Museum (Chicago) or Cleveland Museum of Natural History (Cleveland) for preservation as skin or skeleton research specimens. We focused our analyses on nocturnal migratory passerine birds that collided during the migratory period. Passerine species that are primarily diurnal migrants (e.g. Hirundinidae, Fringillidae and some Icteridae) or residents (e.g. Paridae) represented a small portion of the collision data (3%), as did non-passerines (4%), and were excluded from analyses. Additional details on collision sampling methods (including a discussion of potential biases) and data filtering are provided in the electronic supplementary material.

### (b) Summary of analytical approach

Our objective was to use existing collision monitoring data to test the influence of flight-calling behaviour and habitat characteristics on nocturnal building collision rates, while controlling for relative local abundance, differences in habitat associations, population size and phylogenetic non-independence of species. As described in detail below, we used eBird data [40] to estimate local relative abundance of each species. We performed a *χ*^2^ goodness-of-fit test to estimate deviances in collision tallies of each species from expected numbers of collisions, given these relative abundances. We then tested hypotheses on the role of flight calls and behaviour by (i) modelling the residuals of the *χ*^2^ test as the outcome variable, and (ii) modelling collision counts as the outcome variable directly.

### (c) Collision tallies

In both cities, we tallied the total number of lethal collision records of each species (*n* collisions) and the number of sampling days during which each species was recorded at least once (*n* collision days). We made separate collision tallies for spring and autumn migration for Chicago, whereas we lumped all collision data for Cleveland since that study was only 1 year in duration. Exploratory analyses revealed our results were similar when analysing collision records from McCormick Place separately from the rest of Chicago (electronic supplementary material, figure S5).

### (d) Estimating local relative abundance

As direct estimates of relative species abundance during nocturnal migration are not available, we used diurnal observations from eBird [40] to estimate the relative abundance of each species during migration in Chicago and Cleveland (figure 1; electronic supplementary material, figure S3). Details of how we filtered eBird data are described in the electronic supplementary material. Counting individual birds during birdwatching excursions can be difficult, and accuracy varies widely across observers, species and habitats. Therefore, we considered the number of individuals reported in each eBird checklist to be suboptimal in this context and did not use counts of individuals to estimate the relative abundance of species. Instead, we tallied the number of instances (i.e. checklists) wherein at least a single individual of a species was reported as a proxy for relative local abundance. We used this index (*n* checklists) as the basis of comparison to the number of collisions (*n* collisions). However, the *n* checklists index probably inflates the abundance of rarer species in the eBird dataset (which are sought after by birdwatchers) and may also reduce the abundance of common species that would typically be reported in high numbers in a single checklist. To account for this compression of relative abundance, we repeated analyses described below by comparing a tally of the number of unique days a species was reported in eBird (*n* checklist days) with the number of unique days a species was recorded in collision monitoring (*n* collision days), summed across years. We also included a regional estimate of population size derived from global population censuses as a covariate in our models to account for potential detection bias among species in eBird (electronic supplementary material).

### (e) Flight call categorization

Many decades of study in eastern North America have provided a solid foundation for understanding the basic presence or absence of these behaviours across species [15,16,21,41]. For flight call classifications, we consulted [16] to construct a discrete variable (yes or no) to describe species' typical flight-calling behaviour, and modified certain species classifications based on personal field observations by A.F. (electronic supplementary material, table S1). Two species (*Pipilo erythrophthalmus* and *Tyrannus tyrannus*) give nocturnal flight calls only rarely (A. Farnsworth 2005 and 2018, personal observation). In exploratory analyses, we found that classification of these species as ‘yes’ or ‘no’ for flight-calling behaviour did not affect our results; we classified these two species as ‘no’.

### (f) Habitat classification

We constructed two categorical variables to describe species' ecologies: habitat affinities (forest, edge or open) and typical occupied stratum (ground/low or canopy/upper) with the rationale that species found in the forest understorey may have different visual biology than those of open habitats or canopy [39]. We consulted Birds of North America [42] and considered our own field experience to make these categorizations (electronic supplementary material, table S1).

### (g) Phylogeny

We accounted for phylogenetic relatedness within our models using a 50% majority rule consensus (MRC) tree, calculated from 1000 of the most likely phylogenies from the posterior distribution of a global phylogenetic analysis of birds (http://www.birdtree.org; [43–45]). Branch lengths on the MRC were calculated following [43].

### (h) Collision disparity indices

To compare species’ relative abundances in the collision and eBird datasets, we conducted a *χ*^2^ goodness-of-fit test of species' collision tallies (*n* collisions or *n* collision days), using the relative proportion of each species’ eBird tally of the total tally (*n* checklists or *n* checklist days, respectively) as the distribution of expected probabilities. We performed separate *χ*^2^ tests for the Chicago (spring and autumn) and Cleveland collision datasets.

### (i) Modelling collision disparities

To test the determinants of collision disparities across species in Chicago, we modelled the residuals of each species from the *χ*^2^ goodness-of-fit tests as the outcome variable against the predictor variables of flight calls, habitat and regional population size. We did not perform this modelling of *χ*^2^ residuals for the Cleveland dataset owing to a relative lack of collision data but rather compared the Cleveland *χ*^2^ residuals qualitatively to those from Chicago (electronic supplementary material, figure S6). We first converted the *χ*^2^ residuals to a binary variable based on a standard cut-off of greater than 3 for over-representation and less than −3 for under-representation, excluding species that were found between −3 and 3. This binary variable represents an index of over- or under-representation of species in building collisions compared to their expected frequency in the local species pool (i.e. collision disparity). We used phylogenetically corrected logistic regression [46] as implemented with the phyloglm() function using MPLE in the R package phylolm [47], with 100 bootstrap replicates. We built four models to test the response (residual ∼ flight call, residual ∼ flight call + habitat, residual ∼ flight call + habitat + stratum, residual ∼ flight call + habitat + stratum + regional population size) and compared models using their Akaike information criterion (AIC) scores.

### (j) Modelling collision counts

We also modelled the raw Chicago collision tallies directly as a continuous outcome variable, including the respective eBird tally as one of the predictor variables in a phylogenetically corrected Bayesian generalized linear model implemented in brms [48]. In this model, we used a Poisson distribution because the dependent variable was count data, treated species as a random effect that incorporated phylogenetic relatedness and treated all other predictor variables as fixed effects. We built a simple model to test the response against the eBird tallies with flight calls included as a covariate (collision tallies ∼ eBird tallies + flight call) and model with all covariates included (collision tallies ∼ eBird tallies + flight call + habitat + stratum + regional population size).

### (k) McCormick Place light levels

From 2000 to 2018, D.E.W. and M.H. recorded data on the status of night-time lighting at McCormick Place during pre-dawn walks to collect collisions by recording the proportion of the 17 window bays that were illuminated (figure 1; electronic supplementary material, figure S2). We used this index to test whether building lighting influenced the number of collisions and whether the influence of light levels on collisions counts varied across the sets of species with different flight-calling behaviour or habitat preferences. We tallied the numbers of bird collisions on each day for which a lighting index was available, and modelled collision counts within each flight call and habitat category against the light scores using generalized linear models with a Poisson distribution in R [49]. We also modelled collision counts against light scores using linear modelling by first averaging collision counts across light scores and transforming on a log scale.

## 3. Results

The filtered Chicago collision dataset included 69 785 collisions records of 93 species from 15 families, all of which are small-bodied passerine bird species that migrate predominately at night (electronic supplementary material, table S1). There were 36 315 records from McCormick Place and 33 470 from the remainder of Chicago. The Cleveland dataset includes 2229 collision records from 62 species, all of which were represented in the Chicago dataset.

**Figure 2.**
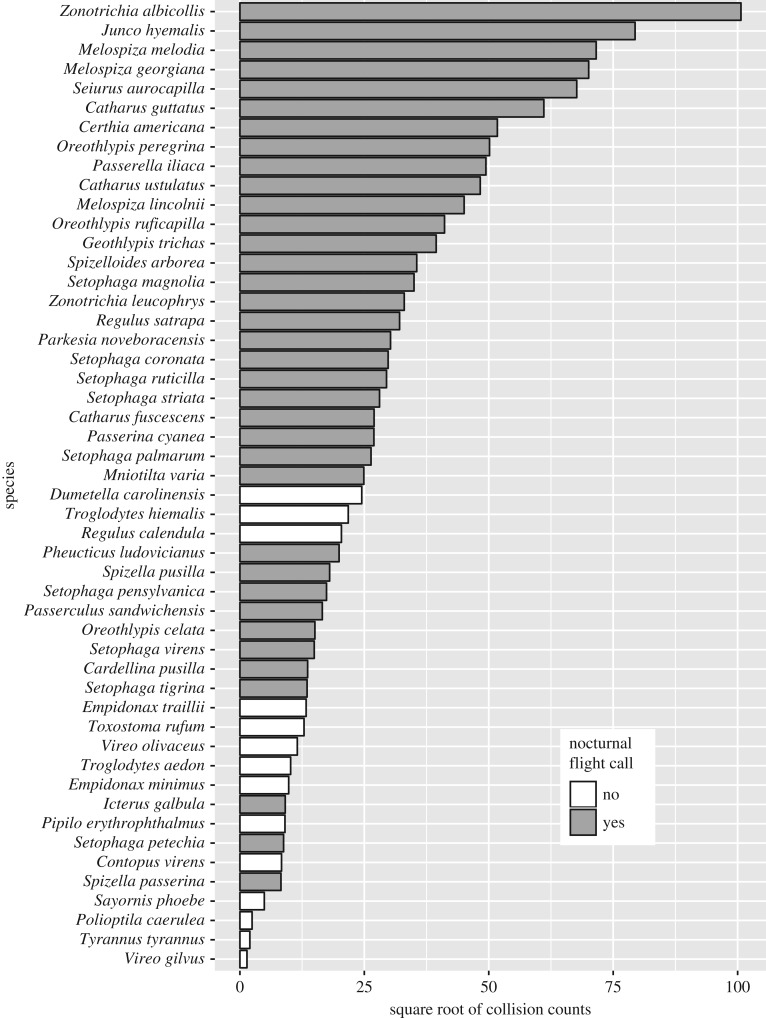
Building collision totals from Chicago for the 50 species most commonly reported in eBird over the same time period (1978–2016). Collision numbers are square root transformed to facilitate visualization; untransformed collision totals ranged from 2 (*Vireo gilvus*) to 10 133 (*Zonotrichia albicollis*). Collision totals of all species in the Chicago and Cleveland datasets are presented in the electronic supplementary material, table S1.

Several species of sparrow (Passerellidae), warbler (Parulidae) and thrush (Turdidae) comprise the majority of our Chicago collision data with thousands of individuals of each species documented as lethal collisions since 1978 (figure 2; electronic supplementary material, table S1). These ‘super collider’ [50] species are all abundant breeders north of Chicago that migrate through the region in high numbers. However, not all locally abundant migratory species are common in our collision datasets (figure 2). For example, in nearly 40 years of monitoring Chicago building collisions, specimens of only two warbling vireos (*Vireo gilvus*), six blue-gray gnatcatchers (*Polioptila caerulea*) and 96 least flycatchers (*Empidonax minimus*) were found, despite their regular occurrence in Chicago during migration—all three species are in the top 25 most reported eBird species in Chicago for the 93 species in the study—and their high abundance in breeding areas north of Chicago [42]. These numbers stand in contrast to more than 10 000 white-throated sparrows (*Zonotrichia albicollis*) collected over the same period (figure 2; electronic supplementary material, table S1). In Cleveland, data from a single year indicate similar taxonomic patterns of collision frequencies as in Chicago (electronic supplementary material, table S2).

**Figure 3.**
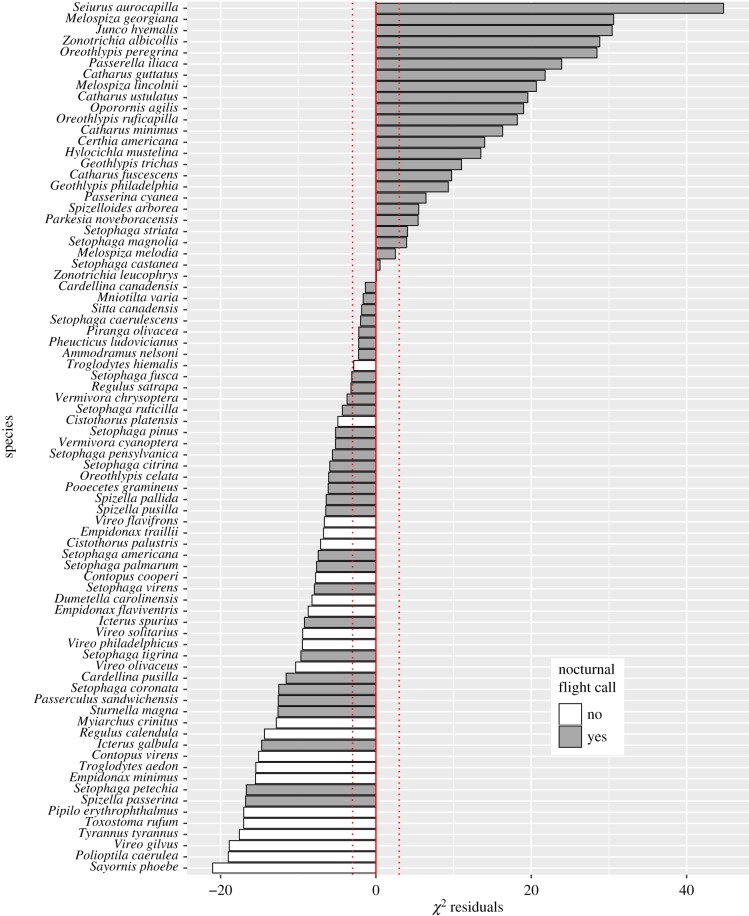
Residuals from the *χ*^2^ goodness-of-fit test of Chicago building collision tallies compared to eBird observations, represented by a tally of unique days that collisions of each species were found (*n* collision days) compared to the proportion of unique days that observations of that species were reported to eBird (*n* checklist days; see Material and methods). Only species with 100 or more eBird checklist days are shown. For visual simplicity, spring and autumn tallies are pooled here but are shown separately in the electronic supplementary material, figure S4, as is the same test using collision counts for each species (*n* collisions) compared to the proportion of unique checklists in which each species was reported (*n* checklists). Negative residuals represent species that are under-represented as collisions compared to expected relative abundance given relative eBird abundance, whereas positive residuals are species over-represented in the collision datasets. Dashed red lines indicate the threshold for defining a binary variable of over- and under-representation in the collision data. All species that are over-represented in the collision dataset make flight calls, whereas species that do not make flight calls are always under-represented in the collision data. (Online version in colour.)

*χ*^2^ goodness-of-fit tests of building collision counts versus relative probabilities of local occurrence in Chicago and Cleveland indicated that all species that are over-represented in the collision datasets (super colliders) use flight calls, whereas all species that do not use flight calls are under-represented in the collision datasets (‘collision avoiders’; figure 3; electronic supplementary material, figures S3 and S4). Our phylogenetically corrected linear models showed that flight-calling behaviour is an important and significant predictor of collision frequency after controlling for phylogeny and relative local abundance in Chicago (table 1; electronic supplementary material, table S3). Statistical support for the importance of flight calling in predicting collision counts was evident regardless of whether habitat variables or a separate estimate of regional population size were included in the models as covariates (table 1). For the generalized linear models of the *χ*^2^ residuals, flight calling was always included in the best fit model based on AIC scores (table 1; electronic supplementary material, table S3). Habitat (classified as forest, edge or open) or stratum (upper/canopy versus lower/understorey) variables were significant predictors in most models but were not always included in the best fit models of the *χ*^2^ residuals (table 1), indicating mixed support for the importance of habitat on collision rates after controlling for phylogeny and population size.

**Table 1.** Results of phylogenetically corrected generalized linear models of a binary collision disparity index as the outcome variable. (This index is defined by the residuals of the *χ*^2^ goodness-of-fit tests of collision data relative to expected abundances derived from eBird data (see Material and methods). Effect sizes are relative to the reference variable. Flight call is a two-state variable (yes/no), habitat a three-state variable (forest, open or edge) and canopy stratum a two-state variable (upper/lower). Regional population size refers to a global population size estimate adjusted for the portion of the breeding range likely to be the source population for Chicago migrants (electronic supplementary material). For data type, ‘count’ refers to *χ*^2^ tests of *n* collisions versus *n* checklists, and ‘days’ refers to tests of *n* collision days versus *n* checklist days (see Material and methods). Significance (italics) codes: **<0.01; *<0.05.)

season	flight call (yes)	habitat (forest)	habitat (open)	canopy stratum (upper)	regional population size	data type
autumn (full model)	3.09**	0.29	−0.67	−1.06	0.13	count
autumn (best model)	3.14*	1.20	−0.91	−2.09**	—	count
spring (full model)	3.39*	−0.10	−0.65	−1.03	0.18	count
spring (best model)	3.04*	0.01	−1.20	−1.25	—	count
autumn (full model)	4.76**	0.86	−0.55	−1.15	0.10	days
autumn (best model)	2.90*	1.82*	−0.45	−2.25**	—	days
spring (full model)	2.79**	0.78	−1.26	−2.05*	−0.12	days
spring (best model)	2.69**	0.78	−1.25	−2.07**	—	days

**Figure 4.**
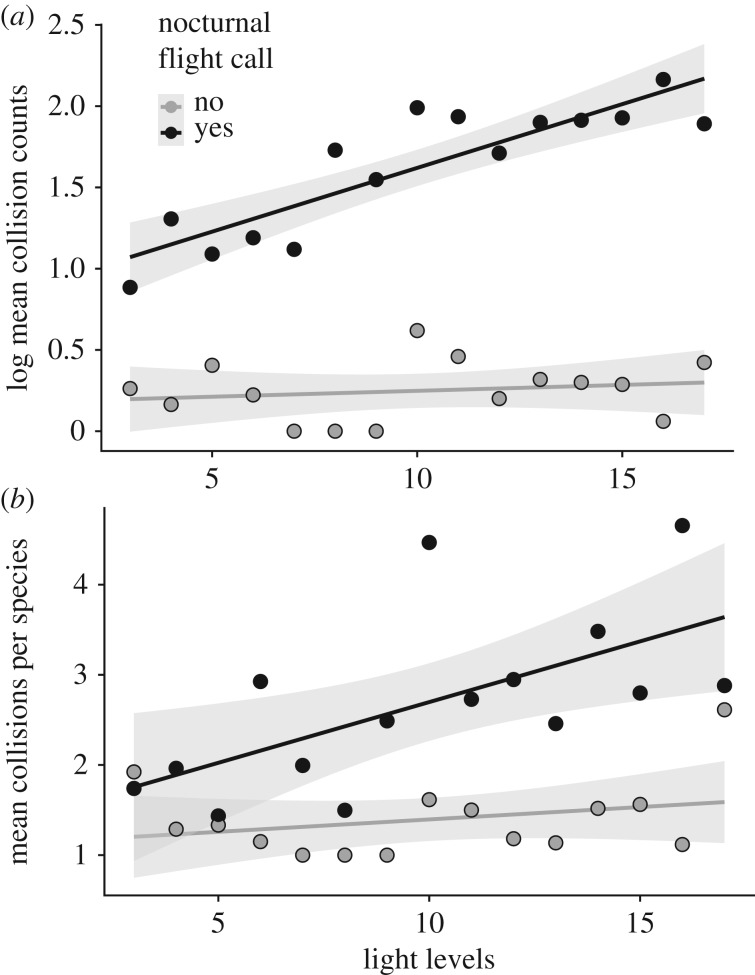
Relationship between light levels at McCormick Place, Chicago and bird collision counts of species with and without flight calls. Light levels are the sum of the number of lighted windows during pre-dawn surveys from 2000 to 2018, ranging as integers from 1 to 17. (*a*) Log mean bird collisions represent tallies of lethal bird collision across all nights for a given light score (see the electronic supplementary material, figure S7), averaged for each light score over the whole study period and log transformed. (*b*) There are more species of flight callers than non-flight callers in the dataset, which could influence the accumulation of collision counts. However, the effect of flight calling is still evident after adjusting collision counts by the number of species in each flight-calling category per night before taking the mean of counts.

Analyses of the extent of artificial light from McCormick Place further suggested that flight-calling behaviour is an important predictor of collision risk, as mediated by differential responses to light depending on whether or not species use flight calls. Within flight-calling species, the number of collisions with McCormick Place on a given night (9381 collisions on 1617 nights for which light scores were recorded) correlated positively with the amount of artificial light originating from the building (figure 4; *p* < 0.001 for generalized linear model of counts or linear model of mean of standardized counts for each nightly light score). Species that do not use flight calls collided less frequently overall (283 collisions over 212 nights for which light scores were recorded), and light levels at McCormick Place had no significant effect on their collision counts (*p* > 0.4 for generalized linear model of counts or linear model of mean of standardized counts). Within flight-calling species, habitat and stratum also had no influence on the relationship between collision counts and artificial light: all classes showed positive, significant relationships of collision counts and artificial light levels (electronic supplementary material, figure S8; *p* < 0.001), though the relationship between open habitat species and artificial light was somewhat weaker.

## 4. Discussion

Our analysis of more than 70 000 nocturnal bird-building collisions from two cities reveals that a primary but poorly understood social cue for nocturnally migrating passerine species—flight calling—strongly predicts species' response to a disruptive environmental stimulus (artificial light). Previous studies found that the rate of flight calling among nocturnal migrants increased around artificial light [27,34], highlighting an interaction between light-induced disorientation and social communication during migration. Our results suggest a related interaction between flight calling and collisions with buildings driven by phototaxis. Although the mechanism explaining this relationship requires further research, we propose that flight calls serve as a social attractant around artificial light, wherein the presence of calling individuals attracts more individuals that produce and use flight calls. When artificial light emanates from a building or other large structure, mortality rates may multiply as more birds are drawn to light. In other words, the principal social cue that may help migrating birds collectively respond to environmental stimuli in situations requiring orientation and navigation instead leads to disproportionately high mortality in an anthropogenically altered world.

There has been speculation as to whether or not nocturnally migrating birds use information from the flight calls of other species [21–23]. The low overall collision rates that we report in species that do not produce flight calls (figures 2 and 3), as well as the weak response of these species to artificial light at McCormick Place (figure 4), in contrast to collision patterns in species that do produce flight calls, provide, to our knowledge, the first circumstantial evidence that the behaviours of species that do not produce flight calls are not influenced by the flight calls of other species. This insight suggests that the use of flight calling is not a trivial idiosyncrasy of natural history but may be representative of broadly different social strategies during nocturnal migration, wherein social interactions during migratory flights are more important for collective decision-making in species that use flight calls than for those that do not. As such, if the presence or absence of nocturnal flight calls across species is indicative of other differences in social behaviour, such as group size and flock cohesiveness during nocturnal migration, then the mechanistic connection between nocturnal flight calls and collision mortality may be indirect. This potential complexity reveals the promise of further research, including the integration of flight call audio recording into collision monitoring efforts, for improving our understanding of the relationship between social biology and attraction to artificial light.

Our results also invite comparative research on the relationship between birds’ physiological and sensory mechanisms for navigation and their social behaviour. Do taxa that rely less on social communication during migration possess or prioritize different sensory systems for navigation and orientation than those that make decisions in part based on cues from their neighbours? Could such putative differences in sensory biology further explain differential responses to artificial light observed between flight callers and non-flight callers? For example, avian disorientation caused by artificial light has been attributed to disruption of a light-sensitive magnetic compass [33,34], but comparative research on this sensory mechanism across avian lineages remains scarce. The insights from our study add a novel behavioural dimension to a rich and rapidly developing literature on the sensory biology of animal navigation during migration [51].

While our results demonstrate that flight calling has a significant influence on collision likelihood, they also reveal unexplained variation in collision rates that raises new questions. We found wide variation in the relationship between local abundance and collision risk across passerellid sparrows and parulid warblers, all of which are known to produce flight calls (figures 2 and 3). For example, yellow warbler (*Setophaga petechia*) and chipping sparrow (*Spizella passerina*) are two abundant flight-calling species that collide with buildings in Chicago infrequently (electronic supplementary material, table S1). More detailed information on calling rates and how flight calls influence collective decisions within and across different species may yet reveal that flight-calling behaviour has even greater explanatory power than detected by our simple presence or absence categorization. However, our results also suggest that habitat association may play a role in predicting collision risk. We found that species of forested and understorey environments tend to collide with buildings in higher numbers than those found in more open environments (table 1; electronic supplementary material, table S3). Yet, lighting levels at McCormick Place did not differentially affect species' collision counts based on habitat associations: species in all types of habitat experienced increased collision rates associated with increased lighting (electronic supplementary material, figure S8). These results suggest that the relationship between habitat and collision risk is probably not owing to habitat-mediated differences in species’ sensory capacities for dealing with artificial light. Rather, we suspect that species of open and edge habitats, even if affected by artificial light, may be more likely to migrate through areas that are further from urban corridors, and therefore collide less frequently, than understorey species of forested habitats. A finer-grained understanding of how species' habitat preferences and stopover ecology during migration influence their collision rates in urban areas will require further research.

Future research should also address potential differences in the biology underlying nocturnal collisions with illuminated buildings versus other structures. Most studies of bird collision risk have focused on assessing population impacts more generally and have typically included data from diurnal collisions (primarily owing to reflective glass) in addition to nocturnal collisions (e.g. [52]). Some of our results are broadly consistent with such studies that have incorporated data from other kinds of collisions, in particular, the widespread collision mortality of thrushes (Turdidae), sparrows (Passerellidae) and warblers (Parulidae). However, for some taxa, our study reveals a contrast between artificial light-driven building collisions and other types of collisions. For example, in studies that have examined bird collisions with communication towers (as opposed to illuminated buildings), vireos (Vireonidae) were more frequent colliders than in this study [29,50].

By focusing our analyses on nocturnal building collisions, our study illuminates striking heterogeneity across avian lineages in the impact of artificial light in urban areas. Yet, despite this variability, our results demonstrate the destructive effect of artificial light for a large number of migratory passerine species (figure 4; electronic supplementary material, figure S6). Although the collision monitoring that yielded the data in this study has led directly to reductions in artificial light at night in Chicago, the problem continues at a large scale in Chicago and other cities [38]. Our results underscore the critical importance for bird conservation of reducing artificial light at night from buildings and other structures during migratory periods.

By revealing the relationship between nocturnal flight calls and building collision rates, our study highlights the importance of evolutionary context for successful collective group decision making: in addition to the potential for group cohesion to correct mistakes [2,53], social cues may provide a mechanism for amplifying individual errors when animals are confronted with novel, disruptive stimuli such as artificial light. Although disruption and mortality from artificial light occurs at a greater scale now than it ever has, light-induced disruption to nocturnal migrants is not purely a function of electricity. On the night of 27 March 1906, the naturalist Witmer Stone observed a large number of nocturnally migratory birds flying low over an enormous lumberyard fire in Philadelphia, drawn lower by the light of the fire. Most birds flew over the fire unaffected, but some flew too close. ‘Up in mid air, apparently clear of flame and smoke, though evidently within range of the terrible heat, a slender thread of silvery smoke came trailing out from the unfortunate bird, like the unfurling of a skein of yarn; it would fly wildly and then, bursting into flame, fall into the roaring furnace below. I saw twenty or thirty birds perish thus during the evening’ [1, p. 251]. Later Stone determined that most of the dead were song sparrows and dark-eyed juncos—two of the species with the highest modern rates of collision owing to artificial light (figure 2). Further comparative research on the interaction between social behaviour, sensory biology and navigation in migratory birds will help elucidate how these animals accomplish their extraordinary journeys and why some lineages are more susceptible to disruption from both natural (e.g. fire) and artificial light sources alike.

## Supplementary Material

Supplementary Information

## Data accessibility

Data available from the Dryad Digital Repository: https://doi.org/10.5061/dryad.8rr0498 [54].
