# Supplementary material for: Nocturnal flight-calling behaviour predicts vulnerability to artificial light in migratory birds
Source: Proc Biol Sci. 2019 Apr 3;286(1900):20190364. doi: 10.1098/rspb.2019.0364 (PMC6501673; doi:10.1098/rspb.2019.0364)
Supplement: Supplementary Information [file rspb20190364supp1.pdf]

Supplementary Information

Winger, B.M., B.C. Weeks, A. Farnsworth, A.W. Jones, M. Hennen & D.E. Willard. Nocturnal flight-calling behaviour predicts vulnerability to artificial light in migratory birds. *Proceedings of the Royal Society B: Biological Sciences*. doi:10.1098/rspb.2019.0364

Supplementary Methods:

Additional Details on Collision Sampling.— Collision monitoring of McCormick Place in Chicago by DEW and MH occurred nearly every morning during the spring and fall migratory periods since 1978, regardless of migration density or weather conditions (data collection is still ongoing, but our analyses included data through 2016). Monitoring of McCormick Place occurred almost exclusively at or just before dawn, ensuring that nearly all specimens salvaged collided during nocturnal migration. Data collected by the Chicago Bird Collision Monitors (CBCM; 2002-2016) and Lights Out Cleveland (2017-2018) was more opportunistic. CBCM has had more than 100 volunteers since 2002 and also receives notifications of dead or injured birds through a phone hotline. The primary purpose of CBCM and Lights Out Cleveland is to rescue live birds and raise awareness about bird building collisions, as opposed to conducting systematic surveys. Monitoring by CBCM and Lights Out Cleveland occurred not only pre-dawn but also occurred opportunistically throughout the day, meaning that some records were of collisions that occurred diurnally due to reflective glass. However, in dense urban areas, artificial light is thought to be the initial attractant that draws nocturnal migratory species towards built areas with reflective glass. Furthermore, exploratory analyses (e.g., Fig. S5) showed that our results are very similar for McCormick Place and the rest of Chicago.

Our data from Chicago consist of records of lethal building collisions, which includes birds that were found dead and those that died shortly after being found. From Chicago, we did not have consistent records of birds that were found alive and later released. All of the species in our study are small bodied passerines (mostly less than 50 grams), and thus differential mortality from collisions is unlikely to have affected our results (exploratory analyses that included body mass as a covariate had no influence on our results). Additionally, the more recent dataset from Cleveland includes both records of lethal collisions and birds that were brought to wildlife rehabilitation centers and released, and in exploratory analyses we found no difference in species composition between lethal and non-lethal collisions within the passerines included in our study. Thus, we treat all collision records from Cleveland the same in our analyses. We also note that scavenging of dead birds by animals such as gulls, crows and raccoons may depress the overall number of collision records retrieved from our study sties, but is unlikely to create a species-specific bias among the small-bodied passerines in this study. Collisions at McCormick Place were retrieved pre-dawn (shortly after collision in most cases), reducing the possibility of scavenging; given that are results for McCormick Place are similar to the rest of Chicago and Cleveland, we do not think scavenging has biased our results.

Filtering of collision data.— We removed all records that did not have a known date of collection, did not occur during the migratory period (March through May and August 10th through November), that were outside the city limits of Chicago (e.g., Evanston, IL), and that were of juvenile or fledgling birds. We also removed 18 records of species whose presence in the collision datasets represented vagrant individuals (i.e. out of range and highly unusual in the study area).

Filtering of eBird data.— eBird data were downloaded for Cook County, IL and Cuyahoga County, OH and filtered geographically to include only the regions in Chicago and Cleveland, respectively, from where our collision data were derived (Fig. S3). To match the collision sampling periods, we included eBird data from Chicago from 1978-2016 (March through May and August 10th through November) and from Cleveland from 2016-2017 (April, May, September, October and November). We included only traveling, area or stationary checklists for which all species that were detected were reported. To limit extralimital and incidental observations, we excluded checklists that were less than 10 minutes or greater than 300 minutes in duration, or greater than 10 km in traveling distance. We reduced "shared checklists" (those shared by multiple observers) to one record. We removed vagrant records and included only nocturnally migratory species from the same taxonomic families as the collision dataset (no families of nocturnal migrants in the eBird data were un-represented in the collision dataset). The eBird data contained records of "*Empidonax* sp.", which are tyrannid flycatchers that are difficult to identify by plumage. Consequently, many sightings are not identified to species, particularly in fall migration. We assigned these records (1,031 from Chicago and 59 from Cleveland) to species based on the proportion of identified records of each species in the datasets (i.e., we assumed that species identity in unidentified records were in the same proportions as identified records). In the collision and eBird datasets, *Empidonax alnorum* and E. *traillii* were lumped as one species because not all specimens were identified to species. The filtered eBird dataset for Chicago contained 175,492 unique observations of 91 species of nocturnal migrants from 16,850 data submission events ("checklists") and for Cleveland 22,510 observations of 97 species from 3,472 checklists.

Regional Population Size Estimation.— We used the North American population size estimates from Partners in Flights [1] corrected by an estimate of the portion of each species' breeding range that likely represents the source population for Chicago migrants. We calculated total range area as the union of a species' breeding and resident ranges, obtained from Birdlife International [2]. For each species, the potential breeding range of the populations sampled in our data was delineated by cropping the total range area to include only likely breeding destinations for birds migrating through Chicago (areas west of the Rocky Mountains, east of Hudson Bay, or in Alaska, were not considered to be part of the potential breeding ranges of individuals passing through Chicago). The area of the potential breeding range of the populations in our data was then divided by the total range area for each species to calculate the percentage of the total population conceivably represented by individuals passing through Chicago, and this fractional estimate of range area was multiplied by the PIF North American population sizes to obtain regional population size estimates for each species.

1. PIF Population Estimates Database (Partners in Flight Science Committee, 2013); http://pif.birdconservancy.org/PopEstimates

2. BirdLife International (2015) IUCN Red List for birds. Available at: http://www.birdlife.org [Accessed January 1, 2015]

Supplementary Tables

Species	Family	Collisions	Flight Call	Habitat	Stratum
Zonotrichia albicollis	Passerellidae	10133	Yes	Forest	Lower
Junco hyemalis	Passerellidae	6303	Yes	Edge	Lower
Melospiza melodia	Passerellidae	5124	Yes	Edge	Lower
Melospiza georgiana	Passerellidae	4910	Yes	Open	Lower
Seiurus aurocapilla	Parulidae	4580	Yes	Forest	Lower
Catharus guttatus	Turdidae	3729	Yes	Forest	Lower
Certhia americana	Certhiidae	2676	Yes	Forest	Upper
Oreothlypis peregrina	Parulidae	2515	Yes	Edge	Upper
Passerella iliaca	Passerellidae	2443	Yes	Edge	Lower
Catharus ustulatus	Turdidae	2331	Yes	Forest	Lower
Melospiza lincolnii	Passerellidae	2029	Yes	Edge	Lower
Oreothlypis ruficapilla	Parulidae	1690	Yes	Forest	Upper
Geothlypis trichas	Parulidae	1555	Yes	Open	Lower
Spizelloides arborea	Passerellidae	1262	Yes	Edge	Lower
Setophaga magnolia	Parulidae	1224	Yes	Forest	Upper
Zonotrichia leucophrys	Passerellidae	1090	Yes	Edge	Lower
Regulus satrapa	Regulidae	1029	Yes	Forest	Upper
Parkesia noveboracensis	Parulidae	916	Yes	Forest	Lower
Setophaga coronata	Parulidae	887	Yes	Forest	Upper
Setophaga ruticilla	Parulidae	868	Yes	Edge	Upper
Catharus minimus	Turdidae	822	Yes	Forest	Lower
Setophaga striata	Parulidae	787	Yes	Forest	Upper
Catharus fuscescens	Turdidae	727	Yes	Forest	Lower
Passerina cyanea	Cardinalidae	725	Yes	Edge	Upper
Setophaga palmarum	Parulidae	694	Yes	Edge	Lower
Mniotilta varia	Parulidae	620	Yes	Forest	Upper
Dumetella carolinensis	Mimidae	599	No	Edge	Lower
Hylocichla mustelina	Turdidae	500	Yes	Forest	Lower
Troglodytes hiemalis	Troglodytidae	474	No	Forest	Lower
Geothlypis philadelphia	Parulidae	430	Yes	Edge	Lower
Regulus calendula	Regulidae	416	No	Forest	Upper
Pheucticus ludovicianus	Cardinalidae	397	Yes	Forest	Upper

Oporornis agilis	Parulidae	365	Yes	Forest	Lower
Spizella pusilla	Passerellidae	325	Yes	Open	Lower
Setophaga pensylvanica	Parulidae	303	Yes	Edge	Upper
Setophaga castanea	Parulidae	287	Yes	Forest	Upper
Passerculus sandwichensis	Passerellidae	274	Yes	Open	Lower
Sitta canadensis	Sittidae	260	Yes	Forest	Upper
Cardellina canadensis	Parulidae	241	Yes	Forest	Lower
Oreothlypis celata	Parulidae	227	Yes	Edge	Lower
Setophaga virens	Parulidae	223	Yes	Forest	Upper
Setophaga fusca	Parulidae	201	Yes	Forest	Upper
Cardellina pusilla	Parulidae	185	Yes	Edge	Lower
Setophaga caerulescens	Parulidae	183	Yes	Forest	Upper
Setophaga tigrina	Parulidae	182	Yes	Forest	Upper
Empidonax traillii	Tyrannidae	177	No	Open	Upper
Toxostoma rufum	Mimidae	166	No	Edge	Lower
Vireo olivaceus	Vireonidae	133	No	Forest	Upper
Piranga olivacea	Cardinalidae	127	Yes	Forest	Upper
Ammodramus savannarum	Passerellidae	106	Yes	Open	Lower
Troglodytes aedon	Troglodytidae	104	No	Edge	Lower
Empidonax minimus	Tyrannidae	96	No	Edge	Upper
Cistothorus palustris	Troglodytidae	91	No	Open	Lower
Icterus galbula	Icteridae	83	Yes	Edge	Upper
Pipilo erythrophthalmus	Passerellidae	82	Rare	Edge	Lower
Setophaga petechia	Parulidae	77	Yes	Edge	Upper
Contopus virens	Tyrannidae	70	No	Forest	Upper
Empidonax flaviventris	Tyrannidae	68	No	Forest	Upper
Spizella passerina	Passerellidae	68	Yes	Edge	Lower
Cistothorus platensis	Troglodytidae	60	No	Open	Lower
Setophaga americana	Parulidae	59	Yes	Forest	Upper
Ammospiza nelsoni	Passerellidae	55	Yes	Open	Lower
Vermivora chrysoptera	Parulidae	53	Yes	Edge	Upper
Spizella pallida	Passerellidae	50	Yes	Open	Lower
Setophaga pinus	Parulidae	49	Yes	Forest	Upper
Myiarchus crinitus	Tyrannidae	28	No	Edge	Upper
Ammospiza leconteii	Passerellidae	25	Yes	Open	Lower
Sayornis phoebe	Tyrannidae	24	No	Edge	Upper
Vireo solitarius	Vireonidae	18	No	Forest	Upper

Geothlypis formosa	Parulidae	16	Yes	Forest	Lower
Vermivora cyanoptera	Parulidae	16	Yes	Edge	Upper
Icteria virens	Icteriidae	15	No	Edge	Lower
Parkesia motacilla	Parulidae	15	Yes	Forest	Lower
Sturnella magna	Icteridae	13	Yes	Open	Lower
Vireo flavifrons	Vireonidae	12	No	Forest	Upper
Vireo philadelphicus	Vireonidae	12	No	Forest	Upper
Pooecetes gramineus	Passerellidae	10	Yes	Open	Lower
Centronyx henslowii	Passerellidae	9	Yes	Open	Lower
Piranga rubra	Cardinalidae	9	Yes	Forest	Upper
Empidonax virescens	Tyrannidae	7	No	Forest	Upper
Icterus spurius	Icteridae	6	Yes	Edge	Upper
Polioptila caerulea	Polioptilidae	6	No	Forest	Upper
Protonotaria citrea	Parulidae	6	Yes	Forest	Upper
Setophaga citrina	Parulidae	5	Yes	Forest	Upper
Contopus cooperi	Tyrannidae	4	No	Edge	Upper
Tyrannus tyrannus	Tyrannidae	4	Rare	Open	Upper
Setophaga cerulea	Parulidae	3	Yes	Forest	Upper
Vireo gilvus	Vireonidae	2	No	Forest	Upper
Zonotrichia querula	Passerellidae	2	Yes	Edge	Lower
Lanius excubitor	Laniidae	1	No	Open	Upper
Passerina caerulea	Cardinalidae	1	Yes	Edge	Upper

Table S1. Species in the Chicago collision dataset, ranked by total number of collisions (spring and fall) from 1978-2016, and their flight call and habitat categorizations.

Taxon	Family	Collisions	Flight Call	Habitat	Stratum
Zonotrichia albicollis	Passerellidae	579	Yes	Forest	Lower
Geothlypis trichas	Parulidae	167	Yes	Open	Lower
Regulus satrapa	Regulidae	166	Yes	Forest	Upper
Melospiza lincolnii	Passerellidae	114	Yes	Edge	Lower
Seiurus aurocapilla	Parulidae	113	Yes	Forest	Lower
Certhia americana	Certhiidae	89	Yes	Forest	Upper
Melospiza melodia	Passerellidae	81	Yes	Edge	Lower
Melospiza georgiana	Passerellidae	76	Yes	Open	Lower
Oreothlypis ruficapilla	Parulidae	70	Yes	Forest	Upper
Setophaga castanea	Parulidae	61	Yes	Forest	Upper
Setophaga magnolia	Parulidae	54	Yes	Forest	Upper
Setophaga striata	Parulidae	51	Yes	Forest	Upper
Oreothlypis peregrina	Parulidae	44	Yes	Edge	Upper
Junco hyemalis	Passerellidae	43	Yes	Edge	Lower
Troglodytes hiemalis	Troglodytidae	34	No	Forest	Lower
Regulus calendula	Regulidae	33	No	Forest	Upper
Catharus ustulatus	Turdidae	32	Yes	Forest	Lower
Dumetella carolinensis	Mimidae	31	No	Edge	Lower
Catharus guttatus	Turdidae	29	Yes	Forest	Lower
Mniotilta varia	Parulidae	27	Yes	Forest	Upper
Setophaga coronata	Parulidae	24	Yes	Forest	Upper
Setophaga americana	Parulidae	22	Yes	Forest	Upper
Geothlypis philadelphia	Parulidae	21	Yes	Edge	Lower
Hylocichla mustelina	Turdidae	16	Yes	Forest	Lower
Setophaga caerulescens	Parulidae	16	Yes	Forest	Upper
Setophaga ruticilla	Parulidae	16	Yes	Edge	Upper
Setophaga palmarum	Parulidae	15	Yes	Edge	Lower
Setophaga virens	Parulidae	15	Yes	Forest	Upper
Cardellina pusilla	Parulidae	13	Yes	Edge	Lower
Setophaga fusca	Parulidae	13	Yes	Forest	Upper
Zonotrichia leucophrys	Passerellidae	13	Yes	Edge	Lower
Passerella iliaca	Passerellidae	10	Yes	Edge	Lower

Setophaga pensylvanica	Parulidae	10	Yes	Edge	Upper
Troglodytes aedon	Troglodytidae	10	No	Edge	Lower
Catharus minimus	Turdidae	9	Yes	Forest	Lower
Oporornis agilis	Parulidae	9	Yes	Forest	Lower
Oreothlypis celata	Parulidae	8	Yes	Edge	Lower
Passerina cyanea	Cardinalidae	8	Yes	Edge	Upper
Pheucticus ludovicianus	Cardinalidae	8	Yes	Forest	Upper
Setophaga tigrina	Parulidae	8	Yes	Forest	Upper
Cardellina canadensis	Parulidae	7	Yes	Forest	Lower
Parkesia noveboracensis	Parulidae	6	Yes	Forest	Lower
Setophaga petechia	Parulidae	6	Yes	Edge	Upper
Spizella pusilla	Passerellidae	6	Yes	Open	Lower
Contopus virens	Tyrannidae	5	No	Forest	Upper
Pipilo erythrophthalmus	Passerellidae	5	Rare	Edge	Lower
Toxostoma rufum	Mimidae	5	No	Edge	Lower
Ammodramus savannarum	Passerellidae	4	Yes	Open	Lower
Cistothorus palustris	Troglodytidae	4	No	Open	Lower
Empidonax minimus	Tyrannidae	3	No	Edge	Upper
Vireo olivaceus	Vireonidae	3	No	Forest	Upper
Catharus fuscescens	Turdidae	2	Yes	Forest	Lower
Geothlypis formosa	Parulidae	2	Yes	Forest	Lower
Passerculus sandwichensis	Passerellidae	2	Yes	Open	Lower
Setophaga pinus	Parulidae	2	Yes	Forest	Upper
Sitta canadensis	Sittidae	2	Yes	Forest	Upper
Spizelloides arborea	Passerellidae	2	Yes	Edge	Lower
Icteria virens	Icteriidae	1	No	Edge	Lower
Icterus galbula	Icteridae	1	Yes	Edge	Upper
Sayornis phoebe	Tyrannidae	1	No	Edge	Upper
Setophaga citrina	Parulidae	1	Yes	Forest	Upper
Vireo philadelphicus	Vireonidae	1	No	Forest	Upper

Table S2. Species in the Cleveland collision dataset, ranked by total number of collisions from 2017, and their flight call and habitat categorizations.

Season	Flight Call (Yes)	Relative Local Abundance	Habitat (Forest)	Habitat (Open)	Canopy Stratum (Upper)	Regional Population Size	Data Type
Fall	**1.66*** (0.76, 2.58)	**1.92*** (1.51, 2.33)	-	-	-	-	Count
Fall	**1.44*** (0.55, 2.32)	**1.71*** (1.18, 2.25)	**1.04*** (0.14, 1.93)	-0.18 (-1.38, 1.02)	**-1.04*** (-1.92, -0.17)	0.16 (-0.34, 067)	Count
Spring	**1.49*** (0.80, 2.18)	**1.50*** (1.19, 1.80)	-	-	-	-	Count
Spring	**1.28*** (0.61, 1.96)	**1.41*** (1.05, 1.78)	**0.76*** (0.10, 1.45)	0.02 (-0.88, 0.93)	**-0.80*** (-1.46, -0.14)	0.13 (-0.23, 0.48)	Count
Fall	**1.36*** (0.67, 2.10)	**1.77*** (1.45, 2.12)	-	-	-	-	Days
Fall	**1.20*** (0.49, 1.94)	**1.77*** (1.34, 2.21)	**0.90*** (0.16, 1.63)	0.15 (-0.84, 1.12)	**-0.77*** (-1.49, -0.04)	-0.10 (-0.50, 0.31)	Days
Spring	**1.20*** (0.66, 1.77)	**1.32*** (1.07, 1.57)	-	-	-	-	Days
Spring	**1.07*** (0.52, 1.63)	**1.30*** (0.99, 1.62)	**0.59*** (0.04, 1.15)	0.11 (-0.64, 0.87)	-0.51 (-1.06, 0.01)	0.01 (-0.29, 0.30)	Days

Table S3. Results of modelling raw collision counts (Count) and the number of days a species collided in Chicago (Days) as a function of species' flight calling behavior, habitat traits, relative local abundance (from eBird) and regional population size. For each season and data type, we estimated a simple model, including only estimates of local relative abundance (from eBird) and the flight calling behavior of the species (present or absent), and a full model, including all predictor variables (see Table 1 caption). Parameter estimates were significant (*) if the 95% confidence interval (given below each parameter estimate) did not overlap with zero. All models included a phylogenetic correction, with species treated as a random effect in a Bayesian generalized linear model (see *Modeling collision counts* in the Methods).

Supplementary Figures

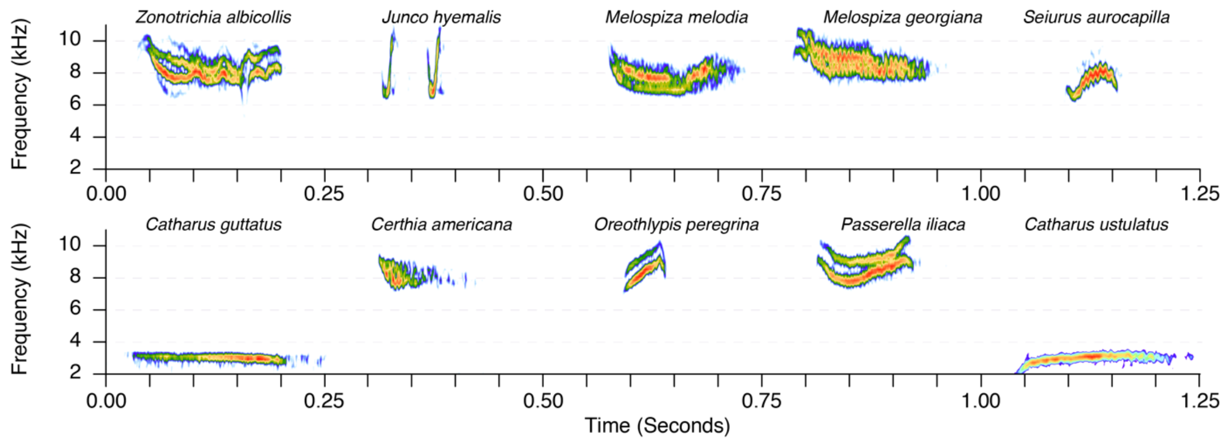

Figure S1. Spectrograms of the flight calls of the 10 species that most commonly collide with buildings in Chicago (Fig. 3). Flight calls are short, high frequency vocalizations characteristic of birds in sustained flight, such as nocturnal migration. Figure courtesy of Kyle G. Horton.

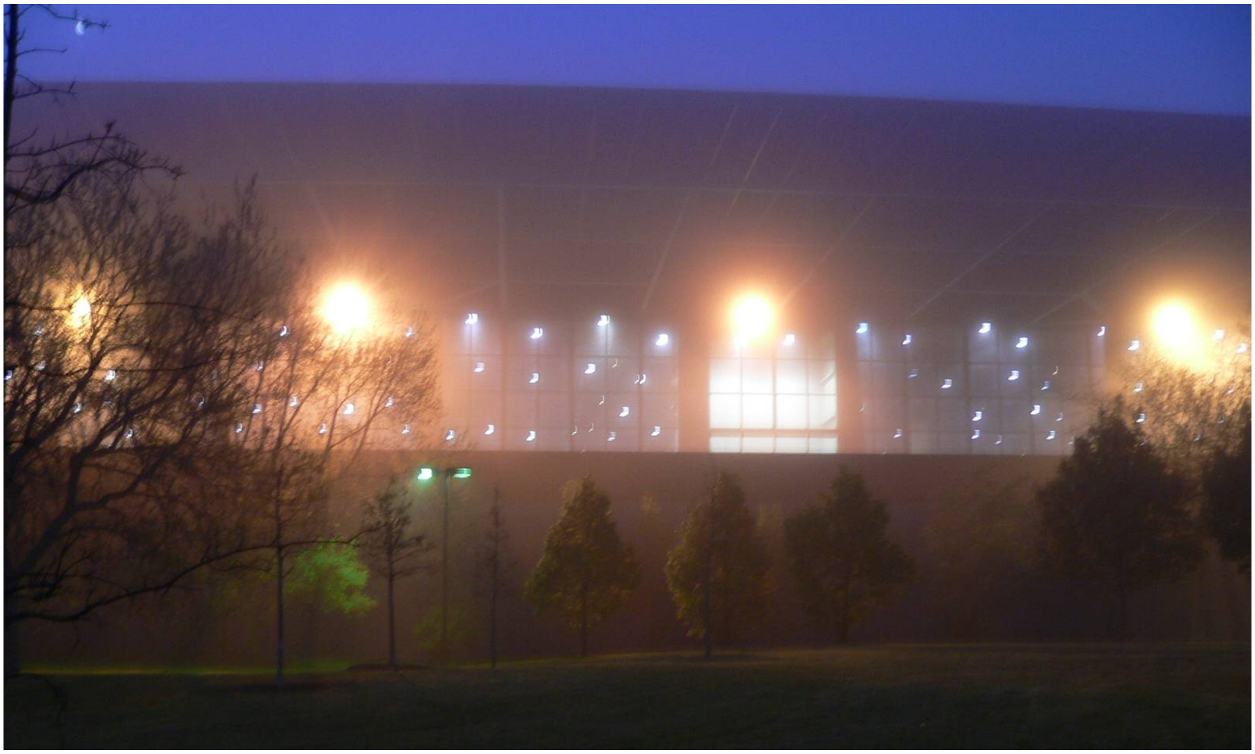

Fig. S2. Illuminated windows at McCormick Place. The windows are recessed beneath a large eave, and lights from within the building illuminate the windows. Photo by David Willard.

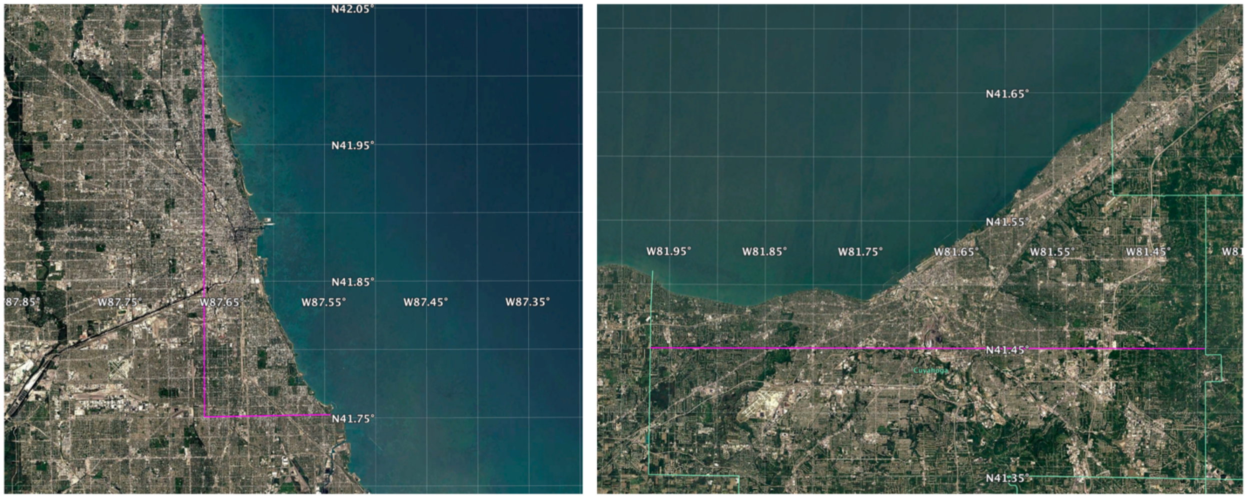

Figure S3. Areas within Chicago (left) and Cleveland (right) from which eBird data were used. For Chicago, we derived eBird data from an area bounded by Lake Michigan, 87.67°W and 41.75°N (pink lines). The western boundary is roughly located along Ashland Avenue and the southern boundary along south 79th street; the northern boundary extends to just south of Evanston, IL. For Cleveland, we used eBird data from an area bounded by Lake Erie on the north, the east and west boundaries of Cuyahoga County (green lines), and 41.45°N (pink line) as the southern border. Both of these bounding boxes contain the densest urban areas where most collisions occurred, particularly along the lakefronts.

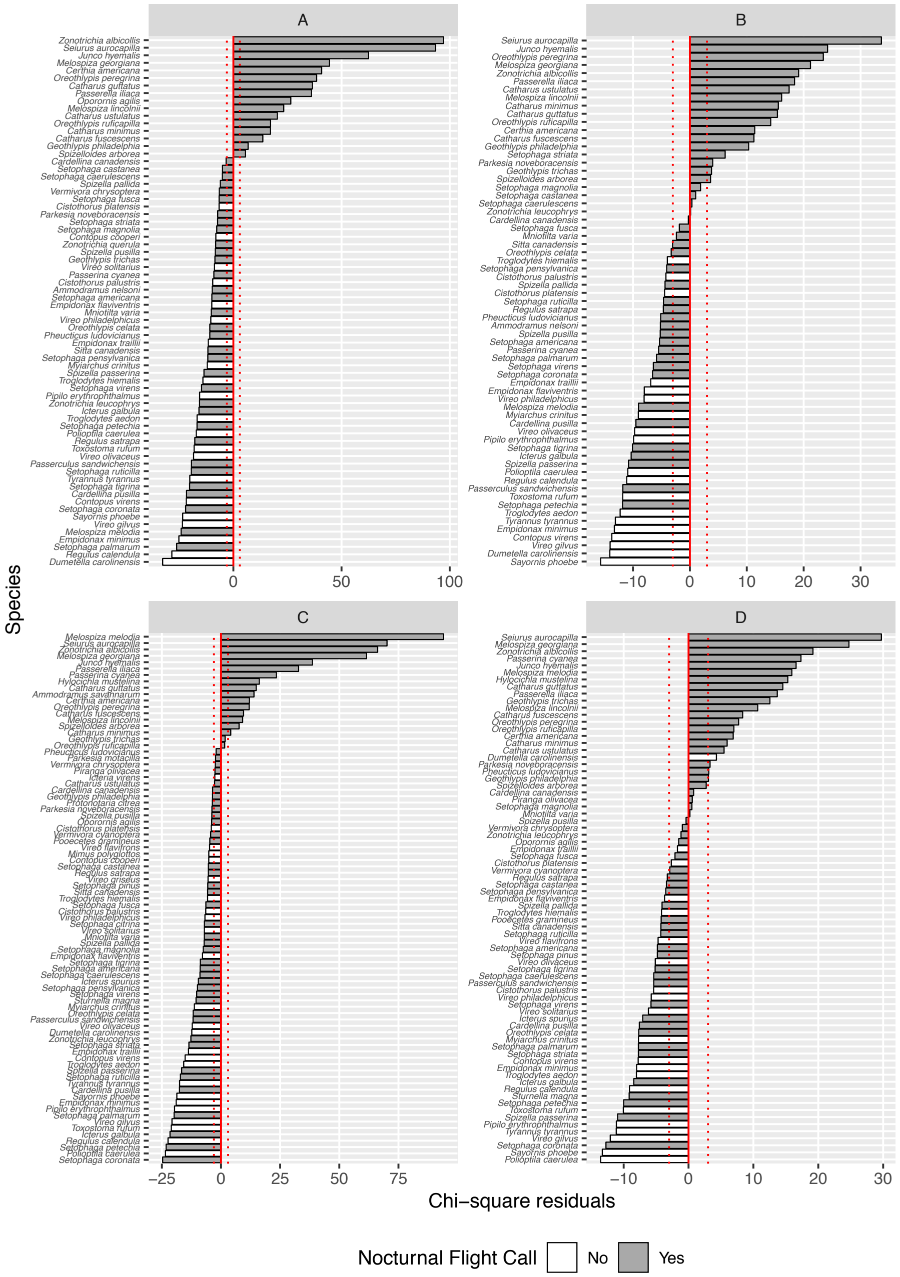

Figure S4. Season-specific residuals from the chi-square goodness-of-fit test of Chicago building collision tallies compared to eBird observations (see Fig. 3 for details). A, C: *n* collisions versus *n* checklists for fall and spring, respectively. B,D: *n* collision days versus *n* checklist days for fall and spring, respectively. Only species with 100 or more eBird checklists or checklist days are shown.

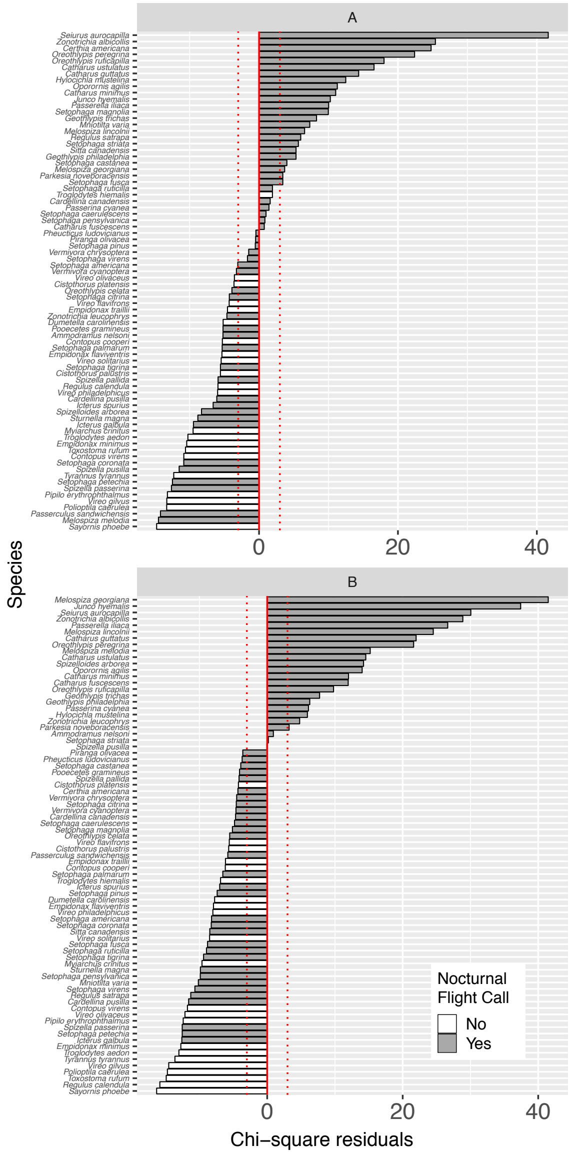

Figure S5. Residuals of chi-square goodness of fit tests of building collision tallies for Chicago excluding McCormick Place (A) and McCormick Place only (B) compared to eBird observations, using *n* collision days versus *n* checklist days with fall and spring combined. Only species with 100 or more eBird checklists or checklist days are shown. The data shown are the same as in Fig. 3 but with collisions separated by the two localities. Exploratory analyses showed that McCormick Place and the rest of Chicago also yielded similar results when separating data by spring and fall, or when using *n* collisions versus *n* checklists (not shown).

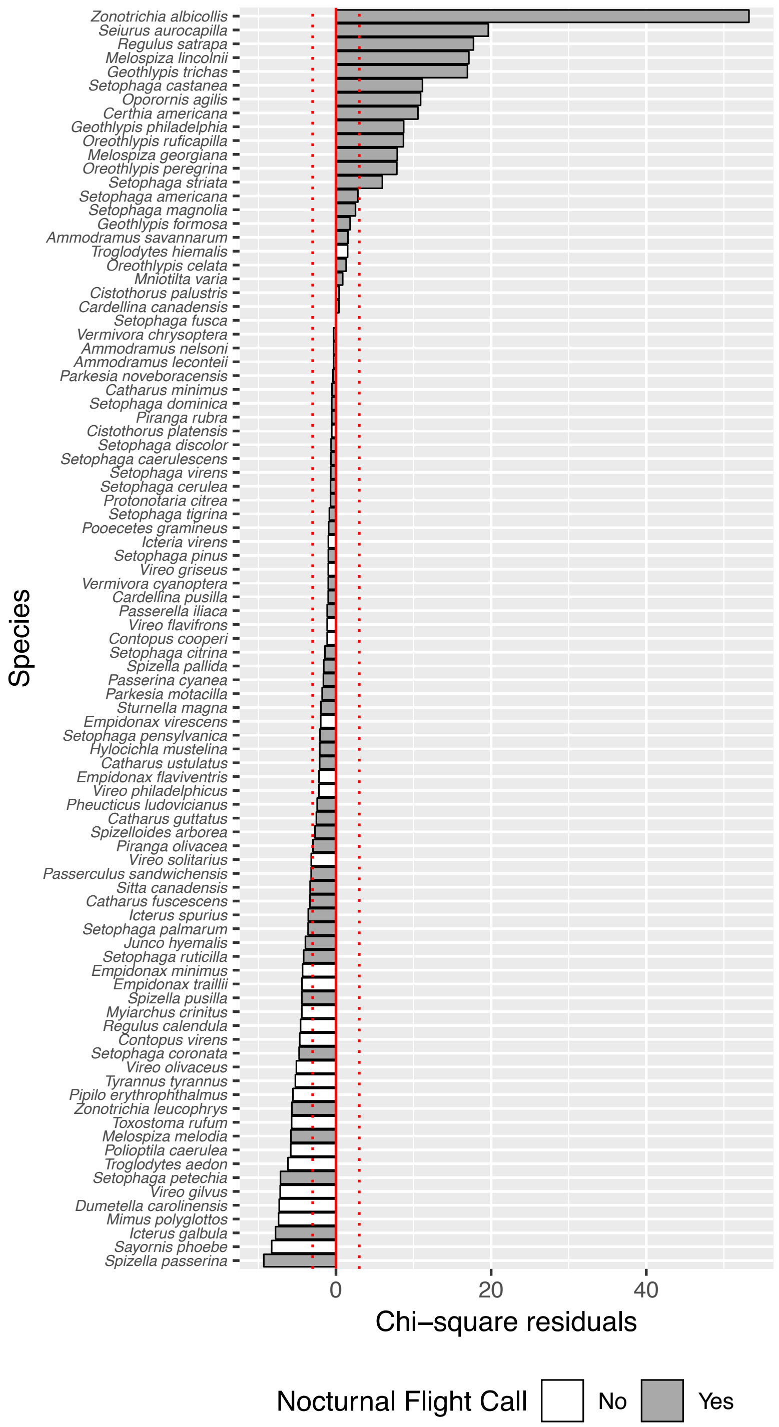

Figure S6. Residuals of the Cleveland chi-square goodness-of-fit test for *n collisions* versus *n checklist.* Only species with 100 or more eBird checklists are shown.

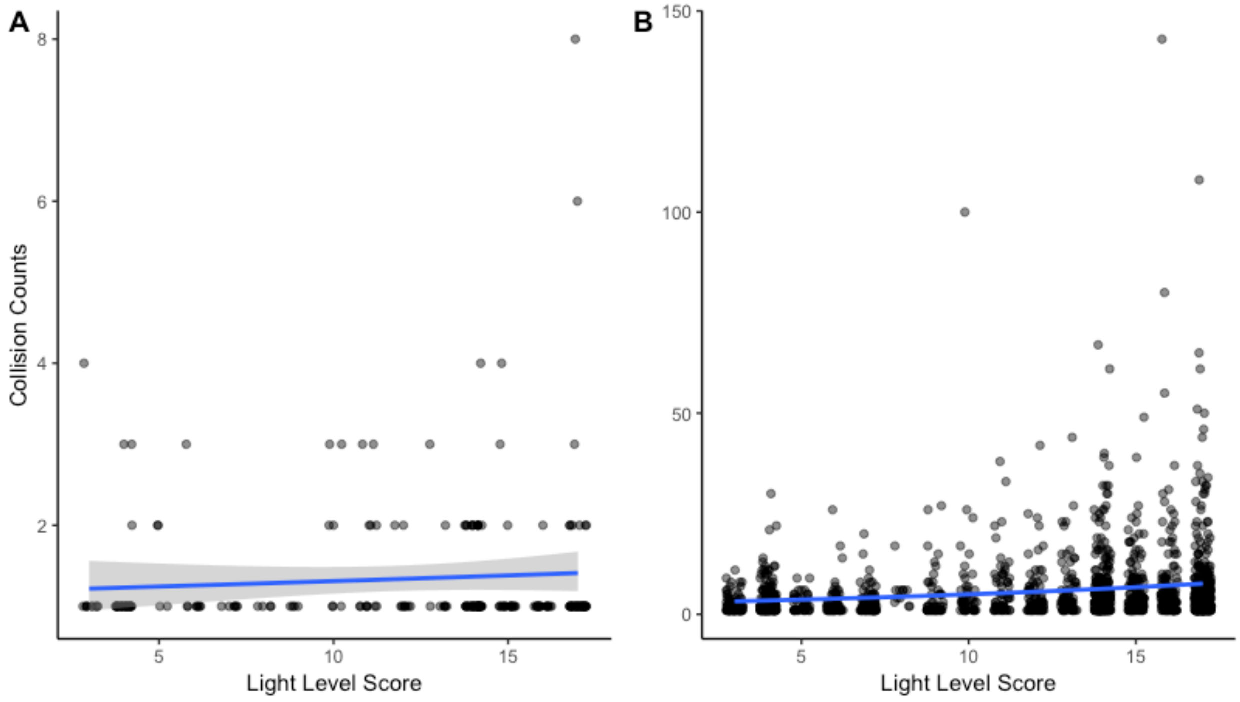

Figure S7. Collision counts for each light score (see Methods for detailed explanation) for A) species that do not make flight calls (283 collisions over 212 nights) and B) species that make flight calls (9,381 collisions on 1,617 nights). Each point represents a single night. Points are jittered horizontally within each score due to over-plotting. Note different scales for y axes. Trend lines represent generalized linear models of the Counts ~ Light Score using a Poisson distribution (see Results). The mean collision counts for each light score are shown in Fig. 4.

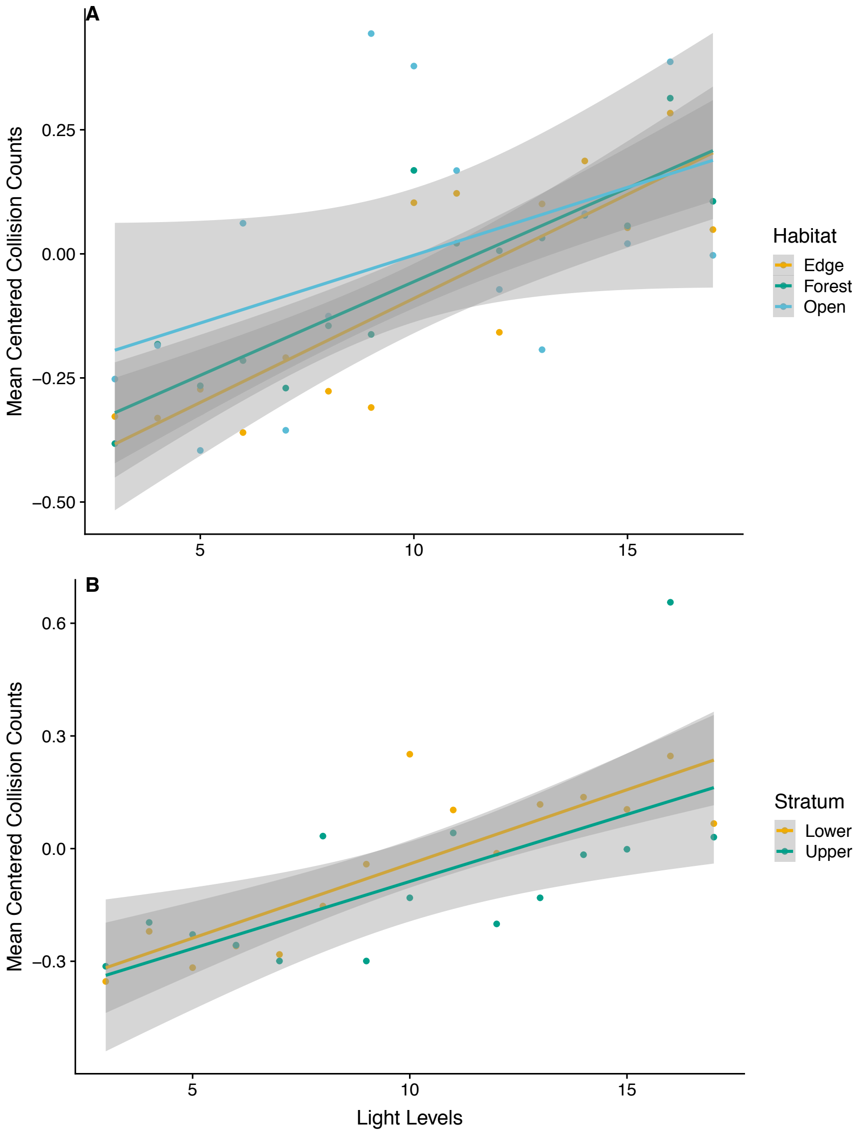

Figure S8. Species in all classes of habitat (A) and stratum (B) exhibited increased collisions with light levels. We standardized collision counts by group mean centering counts within variable categories to facilitate direct comparison of slopes.
